# Supplementary material for: Temporal stability of stimulus representation increases along rodent visual cortical hierarchies
Source: Nat Commun. 2021 Jul 21;12:4448. doi: 10.1038/s41467-021-24456-3 (PMC8295255; doi:10.1038/s41467-021-24456-3)
Supplement: Supplementary file 3 — Reporting Summary [file 41467_2021_24456_MOESM3_ESM.pdf]

nature research

Corresponding author(s): Davide Zoccolan

Last updated by author(s): June 3, 2021

Reporting Summary

Nature Research wishes to improve the reproducibility of the work that we publish. This form provides structure for consistency and transparency in reporting. For further information on Nature Research policies, see our Editorial Policies and the Editorial Policy Checklist.

Statistics

For all statistical analyses, confirm that the following items are present in the figure legend, table legend, main text, or Methods section.

n/a	Confirmed	
☐	☒	The exact sample size (*n*) for each experimental group/condition, given as a discrete number and unit of measurement
☐	☒	A statement on whether measurements were taken from distinct samples or whether the same sample was measured repeatedly
☐	☒	The statistical test(s) used AND whether they are one- or two-sided<br>*Only common tests should be described solely by name; describe more complex techniques in the Methods section.*
☐	☒	A description of all covariates tested
☐	☒	A description of any assumptions or corrections, such as tests of normality and adjustment for multiple comparisons
☐	☒	A full description of the statistical parameters including central tendency (e.g. means) or other basic estimates (e.g. regression coefficient) AND variation (e.g. standard deviation) or associated estimates of uncertainty (e.g. confidence intervals)
☐	☒	For null hypothesis testing, the test statistic (e.g. *F*, *t*, *r*) with confidence intervals, effect sizes, degrees of freedom and *P* value noted<br>*Give P values as exact values whenever suitable.*
☒	☐	For Bayesian analysis, information on the choice of priors and Markov chain Monte Carlo settings
☒	☐	For hierarchical and complex designs, identification of the appropriate level for tests and full reporting of outcomes
☐	☒	Estimates of effect sizes (e.g. Cohen's *d*, Pearson's *r*), indicating how they were calculated

Our web collection on statistics for biologists contains articles on many of the points above.

Software and code

Policy information about availability of computer code

Data collection	The visual stimuli were presented using Psychtoolbox in MATLAB. The neuronal signals were acquired with a TDT system 3 workstation (Tucker-Davis Technologies - TDT) using TDT OpenEx Software Suite.
Data analysis	Neuronal spikes were sorted using the spike sorting package KlustaKwik-Phy. All analyses were performed in Python. The code associated to the analyses presented in this study is freely available at doi:10.17605/OSF.IO/7GTEQ.

For manuscripts utilizing custom algorithms or software that are central to the research but not yet described in published literature, software must be made available to editors and reviewers. We strongly encourage code deposition in a community repository (e.g. GitHub). See the Nature Research guidelines for submitting code & software for further information.

Data

Policy information about availability of data

All manuscripts must include a data availability statement. This statement should provide the following information, where applicable:

- Accession codes, unique identifiers, or web links for publicly available datasets
- A list of figures that have associated raw data
- A description of any restrictions on data availability

The data generated in this study have been deposited on OSF under accession code doi:10.17605/OSF.IO/7GTEQ. The Allen dataset is freely accessible online, as documented in Siegle et al (Nature, 2021). The awake rat data from Vinken et al (Cerebral cortex, 2016), used in this study, are available on OSF under accession code doi:10.17605/OSF.IO/M2E6D

Field-specific reporting

Please select the one below that is the best fit for your research. If you are not sure, read the appropriate sections before making your selection.

- [x] Life sciences
- [ ] Behavioural & social sciences
- [ ] Ecological, evolutionary & environmental sciences

For a reference copy of the document with all sections, see nature.com/documents/nr-reporting-summary-flat.pdf

Life sciences study design

All studies must disclose on these points even when the disclosure is negative.

Sample size	To decide how many neurons to record in each of the areas (V1, LM, LI, LL), we focused on the areas at the top and at the bottom of the hierarchy (V1 and LL) and aimed at recording from them a number of neurons that would allow us to perform linear decoding analyses. Based on existing studies in the literature, we identified this target number as anywhere between 50 and 200, so we settled on 100. In order to account for the anticipated exclusion of neurons due to low reproducibility of firing, we quadrupled this initial number, resulting in a target of 400 well isolated neurons per area in V1 and LL. For the intermediate areas (LM and LI), we collected all neurons we could record in the sessions where we targeted LL. This resulted in the number of neurons reported in the paper: 510 in V1, 126 in LM, 209 in LI and 401 in LL, which was then further reduced when the reproducibility criterion was taken into account (see next point, “Data exclusion”).
Data exclusions	Only those well-isolated units that met a certain trial-by-trial reproducibility criterion were included in the analysis. We excluded all units with reproducibility index lower than 0.7, where the reproducibility index is defined in section “Spike sorting and selection of the units included in the analyses” of the Methods.
Replication	Anesthetized rat data: we did not perform formal replication of our experiments, but all recordings were performed under standardized conditions to ensure reproducibility. Anesthesia, recording, and data processing protocols are described in the Methods, and were followed rigorously.<br><br>Awake mouse and awake rat data: we only used published data for these analyses. We did not perform replication of the experiments that these data sets were collected from. In both cases, detailed protocols for data collection and processing are available in the respective papers.<br><br>Having performed our analyses on three different datasets (anesthetized rats, awake mice and awake rats), collected by three independent labs under equivalent stimulus presentations, each dataset served as a replication of the other two. Specifically, awake mice data in the active wakefulness state replicated the conclusions yielded by anesthetized rat data, while awake rat data under enforced head/body restraint validated the conclusions yielded by awake mice data in the voluntary stillness state.
Randomization	In all three datasets, the order with which movie stimuli were presented was randomized in each session.
Blinding	There was no practical way of blinding the investigators to the identity of the cortical areas of the recorded cells, as determining this identity was a nontrivial part of the initial data acquisition and processing stage.

Reporting for specific materials, systems and methods

We require information from authors about some types of materials, experimental systems and methods used in many studies. Here, indicate whether each material, system or method listed is relevant to your study. If you are not sure if a list item applies to your research, read the appropriate section before selecting a response.

Materials & experimental systems

n/a	Involved in the study	
x		Antibodies
x		Eukaryotic cell lines
x		Palaeontology and archaeology
	x	Animals and other organisms
x		Human research participants
x		Clinical data
x		Dual use research of concern

Methods

n/a	Involved in the study	
x		ChIP-seq
x		Flow cytometry
x		MRI-based neuroimaging

Animals and other organisms

Policy information about studies involving animals; ARRIVE guidelines recommended for reporting animal research

Laboratory animals	19 male Long Evans rats (Charles River Laboratories), age 3-12 months
Wild animals	The study did not involve wild animals

Field-collected samples	The study did not involve field-collected samples
Ethics oversight	Italian Ministry of Health: project N. DGSAF 22791-A, submitted on Sep. 7, 2015 and approved on Dec. 10, 2015 (approval N. 1254/2015-PR)

Note that full information on the approval of the study protocol must also be provided in the manuscript.
